# Using diaries to explore the work experiences of primary health care nursing managers in two South African provinces

**DOI:** 10.3402/gha.v7.25323

**Published:** 2014-12-22

**Authors:** Pascalia O. Munyewende, Laetitia C. Rispel

**Affiliations:** Centre for Health Policy & Medical Research Council Health Policy Research Group, School of Public Health, Faculty of Health Sciences, University of the Witwatersrand, Johannesburg, South Africa

**Keywords:** primary health care, nursing managers, diary methodology, health reforms, South Africa

## Abstract

**Background:**

South Africa is on the brink of another wave of major health system reforms that underscore the centrality of primary health care (PHC). Nursing managers will play a critical role in these reforms.

**Objective:**

The aim of the study was to explore the work experiences of PHC clinic nursing managers through the use of reflective diaries, a method hitherto under-utilised in health systems research in low- and middle-income countries.

**Design:**

During 2012, a sub-set of 22 PHC nursing managers was selected randomly from a larger nurses’ survey in two South African provinces. After informed consent, participants were requested to keep individual diaries for a period of 6 weeks, using a clear set of diary entry guidelines. Reminders consisted of weekly short message service reminders and telephone calls. Diary entries were analysed using thematic content analysis. A diary feedback meeting was held with all the participants to validate the findings.

**Results:**

Fifteen diaries were received, representing a 68% response rate. The majority of respondents (14/15) were female, each with between 5 and 15 years of nursing experience. Most participants made their diary entries at home. Diaries proved to be cathartic for individual nursing managers. Although inter-related and not mutually exclusive, the main themes that emerged from the diary analysis were health system deficiencies; human resource challenges; unsupportive management environment; leadership and governance; and the emotional impact of clinic management.

**Conclusions:**

Diaries are an innovative method of capturing the work experiences of managers at the PHC level, as they allow for confidentiality and anonymity, often not possible with other qualitative research methods. The expressed concerns of nursing managers must be addressed to ensure the success of South Africa's health sector reforms, particularly at the PHC level.

Primary health care (PHC) has re-occupied centre stage in the global efforts towards universal coverage and improved health system performance (1, 2). Within this context, there is global recognition that competent managers are essential for ensuring that priority health needs are met, quality health services are delivered, and that resources are used effectively (3–7). Health managers play a strategic role in planning, allocating resources, and monitoring health policy targets and outcomes (6, 8). At an operational (hospital ward or clinic) level, managers are responsible for effective service delivery (6, 8).

South Africa is on the brink of another wave of major health sector reforms towards universal coverage that underscore the centrality of PHC (9, 10). Nurses are the single largest category of trained health workers, and they play a crucial role in the current provision of PHC services and the management of the existing network of more than 3,000 government PHC clinics and community health centres (11). The PHC re-engineering strategy is one of the major health sector reforms designed to improve population health outcomes and the performance of the health care system (9, 12). These reforms acknowledge the critical role of nurses at the PHC level, both as members of multi-disciplinary clinical teams, and as managers of the community-based outreach teams and school health services (11, 13).

There is a plethora of literature on management and the different conceptual approaches to management (14–18). The management concept has also assumed increasing importance in the health sector (19, 20), with an entire WHO series focusing on different aspects of management strengthening (1, 4, 6, 8). In the health sector, studies have focused on district health managers (i.e. individuals in-charge of an entire health district), and range from a description of socio-demographic characteristics of managers (6), their roles and responsibilities (4, 6, 21), relationships between district managers and their staff, through to assessments of their competencies (4, 6, 21–23). The study findings suggest that there is a general lack of appreciation of managers as a critical component of the health workforce (4, 6, 21). In South Africa, a national assessment of district management structures, competencies, and training programmes found several shortcomings, including incomplete restructuring initiatives, over-extended staff, sub-optimal implementation of policies, and gaps in management competencies (21).

In terms of nurse managers, there have been several empirical studies that have examined the relationship between their management styles and the impact on staff job satisfaction and turnover, patient satisfaction, and quality of care (24–31). These studies have found that transformational and supportive management styles of nurse managers result in lower nurse turnover and higher levels of job satisfaction, which in turn impact positively on patient outcomes (24–31). However, all these studies have concentrated on hospitals, rather than on PHC facilities. In South Africa, there have been a number of studies that focus on nurses working at PHC facilities (32–37). However, the majority of these tend to focus on registered nurses (with 4 years of training) who are the direct service providers (33–35, 37), rather than on PHC clinic managers.

There are several reasons for focusing on PHC clinic nursing managers. Firstly, they are responsible for overseeing the strategic direction of health service delivery, and hence, they play a critical role in the implementation of any health sector reforms (6, 8). Secondly, the literature suggests that effective operational management is positively associated with staff retention, levels of job satisfaction, and quality of patient care (24–31). Thirdly, there is a dearth of information on the perspectives of PHC nursing managers, how they experience or reflect on their work and their practice environment. The aim of this study, therefore, was to explore PHC nursing managers’ work experiences, particularly the successes, challenges or ambiguities faced by them, thereby contributing to recommendations for enhancing management and performance of the health system at the PHC level.

## Research methodology

### Study setting

The diary study was carried out in Gauteng (GP), an urban province, and Free State (FS), a mixed urban–rural province, as part of a larger doctoral study that included a job satisfaction survey (36). These two provinces were chosen due to geographical proximity to the researchers, budgetary constraints, and prior approval from the health service authorities.

### Ethical considerations

The study was approved by the University of the Witwatersrand's Human Research Ethics Committee (Medical), as well as the relevant provincial and municipal health authorities. The researchers adhered to standard ethical procedures, including detailed participant information sheets, informed consent, and ensuring confidentiality of information.

### Population of interest

The population of interest was professional nurses (with 4 years of training) in charge of 8-hour (day) PHC clinics. These clinics serve catchment populations that range from 10,000 to 180,000 (J. Hunter, personal communication, 2014). The clinics provide preventive services (e.g. immunisation, family planning, and antenatal care), basic curative care for acute and chronic conditions, health promotion, and community outreach services.

### Sampling, recruitment, and data collection

During 2012, a sub-set of 22 nursing managers, 10 in FS and 12 in GP, was selected randomly from an overall survey sample of 111 PHC nursing managers in charge of these 8-hour clinics (36). The details of the job satisfaction survey have been described elsewhere (36).

The event-contingent diary method was used, as participants were asked to record an event that answers a specific research question (38). In our study, we were interested in the qualitative experiences of PHC nursing managers – their successes, challenges, and ambiguities – in the workplace. The reason for the selection of diaries over traditional methods such as in-depth interviews was that it enabled the research team to obtain temporal and/or spontaneous information on work events and nursing manager experiences in the PHC clinic context (39). The diaries also allowed for confidentiality and unguarded responses that are not possible with face-to-face interviews.

Each selected clinic nursing manager was given an information sheet and the voluntary nature of study participation was explained to them. Following informed consent, the diary entry guidelines were explained verbally to each nursing manager. The selected clinic manager was then given an attractive diary, with the guidelines pasted in the front of the diary. Participants were assured of confidentiality and asked not to write their names in the diary.

Each manager was requested to write once a week for a period of 6 weeks about an event that happened at the clinic and that stood out for him/her. Once a week was considered reasonable and realistic for nursing managers who work with limited human resources, and it was done to avoid getting limited or no data at all. Participants had to reflect on why they chose that event, how it made them feel, what they learned from it and what the implications were for their current or future management practice. Clinic managers were also asked to write down the date of the diary entry so that these could be counted during analysis. Participants were encouraged to see the diary as their own personal diary, and the researchers undertook to return the diary to them after completion of data capturing. Participant reminders consisted of mobile text messages and weekly telephone calls.

### Data analysis

The diaries were collected from participants and were stored safely at the researchers’ offices in Johannesburg. The diaries were assigned number codes to prepare for analysis and to ensure confidentiality when returning them to their owners. The diaries were also grouped by province to allow for qualitative comparisons.

The diary entries, hand-written in English by each nursing manager, were typed and saved as individual Microsoft Word documents. During data capturing, we noted that diary entries were longer and more detailed in the first week and shorter in subsequent weeks.

The diary entries were analysed using thematic content analysis (40). The first step in the analysis was to look at participants’ own words and phrases and without preconceived notions or classification. We then examined the language used by each participant in light of the following questions: What do the responses tell us about the experiences, feelings and perspectives of PHC nursing managers? What is emerging about the nature and dynamics of PHC nursing management? What is the ‘lived’ reality of PHC clinic managers’ work experiences?

To ensure reliability, two researchers (an experienced qualitative researcher with health system experience and a nurse academic) participated in the development of the themes by reading the diary entries independently from the first researcher in order to establish inter-coder agreement (40, 41). Once the initial analysis was completed, the team met to discuss the themes generated independently, and to reach agreement on the themes and sub-themes (Table 1). Once agreement was reached on the themes, the diary entries were grouped into the various themes (40). Following the generation of themes, a diary feedback meeting was held with PHC clinic nursing managers. They were asked to comment on the themes, whether the themes represented their work experiences, and to reflect on whether the results obtained represented their working experiences. The feedback meeting provided space for reflectivity and ensured the credibility of the research findings. After the meeting, the diaries were returned to participants.

**Table 1:** Diary entry themes

Theme	Description
Health system deficiencies	Complaints about emergency medical services (EMS) Poor referral system Shortages of medicines or consumables
Human resource challenges	Shortages of all categories of staff (e.g. nurses, pharmacists, cleaners) Staff absenteeism Avoidable mistakes by staff, insubordination, or lack of professionalism
Unsupportive management environment	Negative remarks made by clinic supervisor Tension between supervisor and other district-level managers Poor communication (from supervisor or about meetings) Delays in responding to requests for additional staff Failure to honour appointments Demands for health information (monthly statistics, information for research and/or monitoring, and evaluation purposes)
Leadership and governance	Lack of strategic planning Tensions between clinic manager and staff or senior managers Lack of delegation and authority (e.g. of the budget) Difficulties in managing staff or their performance
Emotional impact of clinic management	Feeling scared, tense, being overwhelmed, feeling abused, burnout, exhaustion, frustration, anger, demotivation Includes personal crises at work or at home Patient, community or political complaints about service delivery Perceived burden of urgent or unscheduled meetings Getting positive feedback from clinic supervisors, or feeling supported Sense of achievement or feeling happy

## Results

Diary entries were all hand-written in English. Fifteen clinic nursing managers participated in the diary study, representing a 68% response rate (GP, *n*=10 and FS, *n*=5). The reasons cited for non-participation by the remaining seven clinic managers were: handing over the individual diary to the courier company who lost it (*n*=5), stolen diary (*n*=1) and lost diary during a motor vehicle accident (*n*=1).

The majority of diary participants were female, with only one male respondent. Close to half of the participants were between the ages of 41 and 50 years (45% *n*=10) and a similar number were above the age of 51 (45% *n*=10), and the remainder were in the 21–30 age group. Participants’ work experience ranged from 5 to 15 years. The majority had been qualified as professional nurses for more than a decade and possessed a PHC clinical training qualification.

In general, the participants took a reflective approach in their diary entries. Although inter-related and not mutually exclusive, the themes that emerged from the diary entries were: health system deficiencies, human resource challenges, unsupportive management environment, leadership and governance, and emotional impact of clinic management. All the themes are shown in Table 1 and summarised separately for the sake of clarity.

## Health system deficiencies

Diary entries revealed several health system deficiencies. These ranged from poor emergency medical services (EMS), shortage of medicines, to lack of an enabling environment for service delivery, such as lack of running water. These deficiencies contributed to the difficulties in managing the clinics, as can be seen from the diary excerpts below.

This is not the first [EMS] incident, but it's definitely the worst in terms of time turnaround …. A patient lost her life having waited for more than two hours for an ambulance. Unless the problem is resolved … more patients will complicate or die waiting for an “emergency vehicle.” [Respondent 3, Gauteng Province]

In some clinics in the FS, diary entries show that clinics would sometimes run out of water and this affected the functioning of the clinic. Managers pointed out that hand washing and other infection control measures were dependent on the availability of water. One FS clinic manager had sought support from the clinic supervisor and the municipality but the problem was not being addressed. Eventually, with support from the community, the clinic had obtained a large plastic water container, to serve as a contingency measure for lack of running water. The example below shows the frustration of this FS nursing manager as she wrote about the lack of water as a recurrent problem:

There is no water in the clinic for three consecutive days …. How will you implement infection control and prevention principles when you work without running water for three days? The clinic gets water cuts frequently – almost every 2–3 weeks …. [Respondent 1, Free State Province]

## Human resource challenges

Human resource challenges were the second major theme that emerged, and the diary entries reflected the negative impact on their management activities. Nursing managers documented wide-ranging responsibilities, which include patient consultations, with an apparent disjuncture between their job descriptions and the actual roles they performed in the clinic. Staff shortages impacted on management functions, as managers had to perform clinical duties, in addition to the management functions. In those situations where a staff member was absent or there was a vacant post, nursing managers reported that they had to take on that role, for example, as a pharmacist to dispense medication. High rates of planned and unplanned absenteeism among nursing staff affected clinic operations, and exacerbated the difficulties of PHC clinic management.

I came to work at 7:30 am today realising again nobody came to work. Some are off sick, some just phone to say they will not be coming. Staff shortages are a big problem. I tried to get help again. Nobody from the other clinic can assist. So I must see patients again. All my work is piling up and I did not attend to it yet because of the shortage of staff. Patients are more important than the paper work so I saw patients. [Respondent 7, Gauteng Province]

Staff shortages also led to increased patient waiting times, and in some instances impacted on managers’ health and well-being.

My blood pressure was 156/102 and my glucose level 2.0 mmol/L, I was feeling dizzy and tired. I was unable to go to the doctor because that will mean only one professional will be left at the clinic with more than 100 patients. I never reported the shortage to anyone. The answers that we mostly receive when reporting shortages are “where do you think we will get nurses, find one if you can” that is why we do not report [staff] shortage problems. [Respondent 3, Free State Province]

It was a hectic week, only three nurses on duty on Wednesday and Thursday. I was doing curative [care], adults and children at the same time and I was also busy with statistics in the office. [Respondent 5, Gauteng Province]

The reported staff shortages were exacerbated by perceptions of disabling provincial policies (such as the moratorium on filling posts), staff absenteeism, and an unsupportive management environment.

## Unsupportive management environment

The third major theme that emerged from the diary entries was perceptions of an unsupportive management environment. Clinic managers expressed their disillusionment with their supervisors, who were perceived to be uncooperative and who lacked an understanding of the difficulties faced by them.

My supervisor brought an action plan with time frames. Some of the interventions are not realistic. The clinic was full and staff members were not enough. I was juggling from dispensary, [patient] consulting and solving patients’ minor queries and attending to my supervisor. I feel that I had to give priority to my patients. It was not her [supervisor] visit day according to the schedule. I was disorganised and had to accommodate her …. [Respondent 1, Free State Province]

At times, the diary entries reflected the perceived disrespect, punitive behaviour and verbal abuse from supervisors:

She [supervisor] said there would be no replacement as I only have one entry point in the clinic … she shouted at me that whether I agree or not, she is going to instruct my clerk to go to another clinic which she did … she was so rude and dropped the phone in my ear …. [Respondent 1, Gauteng Province]

On one occasion an FS supervisor did not keep the scheduled appointment, despite calling the clinic manager at her home and giving her instructions for the visit. Nursing managers also complained that clinic supervisors had a top-down approach to supervision and were prescriptive of what needed to be done in the clinics. Supervisors appeared to be unresponsive to requests from clinic managers, especially about additional staff. The excerpt below gives a glimpse of an unpleasant experience of a GP clinic manager.

I had informed the clinic supervisor a month prior to arrange someone for relief [staff] and she had promised to do so. Two weeks before and a week prior, I again reminded her and she still did not know who she was going to send to my clinic to relieve the PHC sister on leave … they sent me someone else whom I was only made aware of that morning. Another professional nurse from the clinic where the relief sister works called demanding that she returns back to her clinic (meanwhile there are four professional nurses in the same clinic) … harassing her that she should return to the clinic. When I checked on her she was tearful and threatening to resign. She found herself torn between wanting to assist at my clinic and being recalled back to her original clinic. This frustrated me even more … the pain … I realised I was … emotionally drained. I called my supervisor who at that time was actually changing from what she said … she now wanted the relief sister to go back to her original clinic while she searched for another one … I refused that the professional nurse leaves the clinic before the relief arrived. After two hours no one arrived. I called again … the nurse was restless and having her bag in hand and was on her way out. This really frustrated me …. [Respondent 2, Gauteng Province]

The above quote reflects the unsupportive approach of clinic supervisors regarding staff shortages and the impact it has on the emotions of nursing managers.

## Emotional impact of clinic management

The multitude of health system problems, human resource challenges, an unsupportive management environment and a range of other problems, coalesced in an overwhelming expression of negative emotions in the diaries, and revealed the emotional impact of PHC clinic management. In some instances, nursing managers wrote about ‘incompetent’ staff reporting to them, and the negative impact on their morale and family life. Importantly, the diary entries reflected the personal stress experienced by these managers at clinics.

I was exhausted … I asked God why I had to come to work with such demotivated staff. I'm starting to hate my work. I know why they are demotivated … they couldn't get study leave, there is no performance management system, even though the population is increasing steadily. It's hard to work with demotivated staff because you must always follow after them for things to be done properly. The thing that hurts the most is that there is no support from co-ordinators of programmes. It's just complaints from patients then staff and from management. Nobody understands the depression we are going through. [Respondent 5, Free State Province]

[My] sleeping patterns are changed because one has to wake up in the early hours of the morning to catch up with administration and [to] meet deadlines. It does not mean one has poor time management but there is a lot of pressure that the manager is subjected to. Family life is also affected by this. Because you come home exhausted, household chores during the week become a challenge. I have sacrificed my weekends and public holidays in order to do my administration. [Respondent 6, Gauteng Province]

PHC clinic managers were frustrated by poor communication from regional and provincial health managers, who often requested information at short notice or summoned them to unplanned meetings. Nursing managers reflected on the perceived burden of these unplanned meetings, despite careful planning on their part. They lamented the lack of control over their average working day as this could be interrupted by ‘an urgent meeting’:

I was very upset on Wednesday. They called me and said there was an urgent meeting and all facility managers must attend. All my plans for the day messed up. [Respondent 12, Gauteng Province]

We were called for an urgent meeting whereby one of our colleagues was together with the supervisor questioning our Regional Health Manager's authority to delegate authority to us as operations managers. Assessing the whole deliberation, I realised that we were caught up in an ongoing misunderstanding and poor communication between the two senior managers … it causes paralysis …. [Respondent 9, Gauteng Province]

Despite the experience of negative emotions caused by an unsupportive management environment, health system deficiencies and unplanned meetings, clinic managers recognised their important role in health service delivery. They reflected on their responsibilities of: implementing health programmes in the clinic, managing human resources, liaising with community members and relevant stakeholders, and ensuring that clinic operations run smoothly. Notwithstanding the challenges experienced by nursing managers, the diary entries suggest nursing managers who have great concern for patients and the quality of care delivered. One made the following diary entry:

On that morning, the clinic was so full and there were many babies for immunisation and sick adults in the main hall. The passage leading to my office was packed! I had to ask 13 antenatal clients (three new cases) to wait inside the small fourth consultation room. I had to attend to family planning clients and to ARV initiation clients who need to be assessed and have their bloods taken for baseline, to TB patients who were collecting their medication and also referring one very sick (TB/HIV) patient which took almost an hour. [Respondent 1, Gauteng Province]

My “little voice” told me to check the BP (blood pressure) again – it was 240/160!! Severely, severely raised! Apart from now having to treat and refer a pre-eclamptic patient I also realised the terrible risk we take by relying on vital signs taken by a nursing assistant. [Respondent 2, Free State Province]

Despite their crucial role at the PHC level, nursing managers indicated that they seldom receive positive feedback or feel appreciated in the health system. Three entries showed the appreciation of clinic managers when they received positive feedback from their managers or when they felt a sense of achievement:

It was a clinic managers meeting where we were given feedback on programme performance for each clinic. I was told that our tuberculosis (TB) programme had improved since I allocated two professional nurses with the intention of making the programme a success. [Respondent 13, Free State Province]

I came on duty in my culture day dress and it was very nice. No problems this far. Two nurses did not pitch for work but clinic was not that full so I can do my work. I worked out the off duties and started on my report. It was a lovely day and I got all my things done. [Respondent 12, Gauteng Province]

I had local area meeting on Wednesday. I am feeling good because the manager mentioned that our clinic does the best we can with limited resources (staff). I am just glad she realises it. [Respondent 1, Free State Province]

## Leadership and governance

Some nursing managers reflected on feelings of disempowerment, and at times ‘paralysis’, caused by the lack of strategic planning at higher levels of the health system and the difficulties of managing staff reporting to them, the absence of teamwork and their perceptions of a general lack of caring and professionalism on the part of frontline nurses. They complained of the ‘poor work ethic’ among many nurses reporting to them, changing value systems, resistance to change, and lack of accountability. They also reflected on the importance of leadership in nursing given the constant changes in the healthcare system.

However, the diaries revealed that nursing managers do not hold the chain of command in clinics as this power resides with the clinic supervisor and in most instances with the district manager. The local area manager, who oversees several clinics, is responsible for the clinic budget. PHC clinic managers bemoaned the centralisation of the clinic budget, and their lack of control thereof, despite their responsibilities for the day-to-day management of the clinic. The clinic managers were often not consulted on spending priorities, despite compiling annual budget requests. One said the following:

The budget is centralised, one has no power over it. The financial year comes and goes with little improvement … shortages of medicines occur because suppliers are not being paid. [Respondent 1, Gauteng Province]

Clinic managers also receive instructions from doctors, pharmacists, social workers, and vertical health programme co-ordinators responsible for HIV or tuberculosis. All these combine to add further pressure on the clinic manager.

## Discussion

This is one of the first studies to explore the work experiences of PHC nursing managers in two South African provinces using diaries as a research method. The major recurring themes in the diary entries were health system deficiencies, human resource challenges, and an unsupportive management environment – these problems are inter-related and contributed to the difficulties of working in or managing these PHC clinics.

PHC clinic managers expressed frustration with EMS problems and the unpredictable turnaround times, which in one case resulted in a seemingly avoidable patient death. Reliable EMS services have been found to be a critical component of health systems strengthening (42). The nursing managers both reacted and responded to the health system deficiencies in their own way, either by trying to cope with staff shortages or by responding creatively to the lack of water in rural clinics, through partnering with the local community. Other studies have also found that PHC clinic managers often balance operational management and service delivery to many patients amidst staff shortages in the health system (33–35). Although the diary entry on the lack of running water in some FS clinics appears to be an isolated incident, the lack of running water at rural clinics is a common finding in national infrastructure assessments (43). This influences the ability of nurses to comply with infection control standards in these rural clinics, and contributes to the sub-optimal performance of the health system.

The issues highlighted in the diaries resonate with health system deficiencies found in other studies (33, 35, 44, 45). Staff shortages were highlighted in all diaries. The factors that appear to influence these shortages included provincial policies (such as a moratorium on filling of vacant posts), inadequate or poor planning on the part of clinic supervisors, and absenteeism of frontline staff. This meant that PHC nursing managers had to take responsibility for clinical duties often at the expense of their administrative or managerial duties. Although this diary study was small and qualitative, other studies have found that staff shortages have constrained South Africa's ability to achieve the strategic planning goals on HIV and AIDS (46) and the implementation of the services at PHC level (34, 47).

The reported staff shortages were made worse by nursing managers’ perceptions of largely unsupportive supervisors. PHC nursing managers wrote about the lack of understanding, disrespect and at times verbal abuse from their supervisors. Notwithstanding the existence of the detailed clinic supervision manual (48), there appears to be a disjuncture between the supervision guidelines in the manual and the clinic managers’ diary reflections of an unsupportive management environment. For example, the manual states that: ‘for the best provision of PHC in facilities, there should be a supervisor who facilitates good teamwork and promotes good working relationships among all the structures of the primary health care system’ (48, p. 4). The lack of quality clinic supervision has been found in other studies as well (49, 50). Effective supervision of PHC clinics is a critical issue that needs to be addressed, given that health sector reforms include a wide range of community-based services and the inclusion of community health workers (47).

In light of the reported challenges experienced by PHC nursing managers, it is not surprising that the diary entries were dominated by an expression of negative emotions, which could be a symptom of the stress experienced by these managers. In response to the question on how the recorded event made them feel, the most frequent responses were: exhausted and frustrated, angry, sad, burnt out, and demotivated. This was borne out by the larger job satisfaction survey, which found that being tired at work and the experience of verbal abuse were predictors of low job satisfaction of these nurses (36). A study in Lithuania among PHC nurses also found that around 60% of nurses experienced negative emotions and resultant emotional stress (51). The Lithuanian study further found that bullying and abuse by supervisors in the workplace caused stress and contributed to feelings of humiliation and disrespect (51). Similarly, a study in Taiwan found that 25% of nursing managers were depressed: 30% suffered from anxiety anxiety and 44% suffered from poor quality of sleep leading to high levels of burnout and lower rates of retention (52). Despite some of the negative emotions and experiences recorded in the diaries, overall, the entries reflect a commitment to providing quality care and a need to be acknowledged for their hard work.

There are limitations of this diary study, which was undertaken among a sub-sample of 22 PHC nursing managers. The majority of study participants were from GP, which is the economic powerhouse of South Africa. These clinics are likely to be much better resourced, compared to deep-rural clinics in other parts of South Africa. We had fewer diaries from the FS Province, due to the logistical problems experienced with the courier company. Hence, the study findings may not be transferrable to other PHC clinics in South Africa or elsewhere.

Nonetheless, the findings from the diary study are borne out by the findings of national health system assessments which have highlighted health system deficiencies, human resource challenges and supervision and management problems at the PHC level (44, 45). The diaries are an innovative method of capturing the nature and dynamics of nursing management, as the method allows for confidentiality and anonymity, often not possible with individual interviews or focus group discussions. The diaries gave a voice to PHC nursing managers, facilitated greater self-awareness and allowed them to reflect on their management practices. Nursing managers reported that the diaries were cathartic, as it allowed them to say things that no-one in authority could see or hear. In some instances, the diaries facilitated practical action with identified problems at PHC level, such as when one nursing manager communicated directly with the senior EMS manager after a patient had died. However, the use of diaries requires participant commitment and buy-in, as well as good preparation and initial piloting prior to implementation. It is important to ensure that study participants understand the study objectives and the guidelines for keeping a diary. The success of diary studies depends on regular communication with participants through constant reminders to ensure compliance and maintain the interest of the respondents.

The diary entries have given a glimpse into the difficulties of policy implementation at the local level, from the perspective of PHC nursing managers. These managers give effect to high-level government policies as they are at the interface of community members (and patients) and the formal health system. The PHC nursing managers are expected to manage the bulk of PHC reforms. Their experiences of disempowerment and paralysis need to be addressed through a participatory and inclusive approach, which could simply mean eliciting their views and opinions regarding prerequisites and implementation strategies. This is important because they have to mediate or manage complex health system problems, while ushering in the proposed reforms.

The human side of the managers found expression in a deluge of negative emotions recorded in the diaries. This study has shown that relationships matter and that how they are managed has an impact on how services are delivered or managed. The diary study has also illustrated the resilience among PHC nursing managers and their strategies for coping with a sub-optimal health care system in order to provide adequate care to patients or users. Inflexible hierarchies or policies (e.g. around staff recruitment) appear to make clinic work more onerous, with potential negative consequences for patients and clinic managers. Nursing managers are also curtailed by the centralisation of budget control, and they have to rely on supervisors who do not seem to know how to communicate effectively with them. This lack of delegation of authority, particularly of the clinic budget, exacerbated the reported health system deficiencies. The sense of disempowerment and paralysis experienced by PHC clinic managers was illustrated by the many negative emotions recorded in all the diaries. The relationship between the inability to manage or control the budget and feelings of disempowerment was also found in a 2008 assessment of district managers (21).

Although this was a small, qualitative study, the realities experienced by nursing managers point to issues that need to be addressed as part of the universal health coverage reforms in South Africa. Firstly, efforts to improve the performance of the health system must be comprehensive and recognise that PHC revitalisation must be accompanied by effective and efficient EMS, and appropriate delegation of authority. Secondly, chronic staff shortages require creative strategies, and there appears to be room for improved performance management to reduce staff absenteeism. Thirdly, there are clear guidelines for supportive clinic supervision, which appear to be largely ignored at present. Supervisors may need to be reoriented to the guidelines or receive additional training to enhance their supervision skills. Clinic managers have long experience in the health services, and the health system needs to find a way of harnessing their wisdom in support of current health reforms. Lastly, the identified challenges need to be addressed by policy-makers working together with managers at all levels of the health system, given that health system reforms will create different work demands and diverse experiences for nursing managers.

## Conclusions

This study has highlighted the work experiences of PHC nursing managers, using diaries, a hitherto under-utilised research instrument. The PHC clinic managers’ negative emotions expressed in the diaries have the potential to affect or derail health system reforms, as demoralised PHC nursing managers are unlikely to be champions for change or be committed to such change. At the same time, the PHC nursing managers who participated in the study highlighted the importance of sufficient numbers of health workers, supportive supervisors, and optimal functioning of the health system. The current reform process of South Africa's healthcare system provides a golden opportunity for policy-makers to address the root causes of health system inefficiencies in a participatory manner and through the creation of enabling work environments. To this end, the critical role of the health workforce requires much more attention than is currently the case. Addressing the challenges identified in the work experiences of PHC nursing managers would go a long way in ensuring the successful implementation of health sector reforms.
